# A Novel Technique for Eye Rejuvenation: A Case Series of the Combined Use of CO_2_ Laser Blepharoplasty and Erbium: YAG Resurfacing and A Novel Artificial Intelligence Model to Quantify Laser Results

**DOI:** 10.1111/jocd.70022

**Published:** 2025-02-07

**Authors:** Chelsea E. Kesty, Katarina R. Kesty

**Affiliations:** ^1^ St. Petersburg Skin and Laser St. Petersburg Florida USA; ^2^ Kesty AI St. Petersburg Florida USA

## Abstract

**Background:**

Consumers are searching for a solution to rejuvenate the eye area. Surgical blepharoplasties are a common solution, but they lack improvement in skin quality.

**Aims:**

To present a novel procedure of a laser upper blepharoplasty in combination with erbium laser resurfacing of the lower eyelid for optimal rejuvenation and minimal complications.

**Methods:**

The authors present a laser upper blepharoplasty with the CO_2_ laser performed at the same time as lower eyelid resurfacing using an erbium laser. The authors used an artificial intelligence large language model to assess the patient before and after photographs to quantify cosmetic improvement.

**Results:**

After this novel procedure, patients demonstrated significant improvements in upper eyelid contour, reduced skin laxity, and smoother lower eyelid texture. Patient satisfaction was high, with each patient reporting an overall rejuvenated appearance and a more “awake” and youthful look. The artificial intelligence algorithm showed cosmetic improvement in line with the clinical evaluations by the patient and physician.

**Conclusions:**

The combination of CO_2_ laser blepharoplasty and Er:YAG laser resurfacing addresses both upper eyelid dermatochalasis and lower eyelid wrinkles effectively while minimizing recovery time and the potential for complications. Artificial intelligence models were used to enhance this study and corroborate evaluator cosmetic improvement.

## 1 Introduction

Seventy‐nine percent of consumers are looking for a solution to treat the lines and wrinkles around their eyes [1]. Eye blepharoplasties are popular cosmetic procedures that have increased by 13% over the last few years [2]. Periorbital rejuvenation remains a critical focus in aesthetic facial procedures, as the eye area is often one of the first to show signs of aging and one of the most popular reasons to seek a cosmetic procedure.

The CO_2_ laser has been widely recognized for its precision and hemostatic properties, making it ideal for delicate upper eyelid surgeries. Compared to traditional blepharoplasty, CO_2_ laser blepharoplasty minimizes intraoperative bleeding, reduces postoperative bruising, and allows for fine control over tissue excision. Studies have demonstrated that CO_2_ lasers provide durable results in upper eyelid rejuvenation, with a lower incidence of scarring and complications compared to other modalities [3, 4, 5, 6].

While CO_2_ lasers are effective for deeper ablation, their intense thermal effects may increase the risk of hyperpigmentation and scarring, especially in lower eyelid treatments [7, 8, 9, 10]. Er:YAG lasers, with their shorter wavelengths, are ideal for controlled, superficial resurfacing [11, 12]. They have a low risk of thermal damage, making them especially suitable for delicate lower eyelid skin [13, 14]. Research indicates that Er:YAG lasers are highly effective in reducing periorbital wrinkles and tightening skin with a shorter recovery time and fewer side effects than CO_2_ lasers [12, 14].

The use of laser‐assisted techniques for upper eyelid blepharoplasty and lower eyelid resurfacing has gained traction due to their minimal invasiveness and precision [4]. Carbon dioxide (CO_2_) lasers and erbium‐doped yttrium aluminum garnet (Er:YAG) lasers offer unique benefits in periorbital rejuvenation, particularly when used in tandem. CO_2_ laser blepharoplasty enables precise tissue ablation and effective hemostasis, which are essential in delicate periorbital anatomy. Er:YAG resurfacing, on the other hand, minimizes thermal injury in lower eyelid resurfacing, which is advantageous in reducing complications like hyperpigmentation and extended recovery periods [13]. This report presents a case series where CO_2_ laser blepharoplasty on the upper eyelids was combined with Er:YAG resurfacing on the lower eyelids, examining the outcomes of this approach. This is a novel technique that has not been described in the literature.

The use of artificial intelligence in dermatology is limited and has yet to be used to quantify results of lasers. One potential use of artificial intelligence is to use a model instead of physician evaluators to standardize results of lasers and other cosmetic procedures.

## 2 Methods

This case series involves 10 patients (aged 40–73 years, six female, four male) who presented with concerns of eyelid aging, including dermatochalasis of the upper eyelids, periorbital wrinkles, and skin laxity of the lower eyelids. Each patient sought minimally invasive treatment to improve eyelid aesthetics without undergoing traditional surgical blepharoplasty. They were in good general health and had Fitzpatrick skin types I–III, making them suitable candidates for ablative laser procedures. The authors used an artificial intelligence large language model to assess the patient before and after photographs (Kesty AI, St. Petersburg, FL). All 10 patient pre‐operative and post‐operative photographs were put into the artificial intelligence model (a total of 20 photos). The artificial intelligence large language model was designed to output Fitzpatrick Wrinkle Scale and Glogau Wrinkle Scale based on the patient photographs of their face. We used this artificial intelligence model to determine the change in the patients' scores on these two scales to quantify results of this laser procedure.

For preoperative preparation, each patient underwent a comprehensive ophthalmologic examination to ensure eyelid and ocular health. Informed consent was obtained after explaining the risks, benefits, and alternatives. For anesthesia, a local anesthetic (lidocaine 1% with epinephrine) was administered along the upper and lower eyelid areas. Topical anesthetic cream was applied to the surrounding periorbital skin.

For the blepharoplasty, a pulsed CO_2_ laser with 10,600 nm wavelength (Ultrapulse, Lumenis Be Ltd, Israel) was used to incise and excise excess skin on the upper eyelids. This technique allowed precise tissue ablation with minimal blood loss due to the CO_2_ laser's coagulative effects. Each upper eyelid was carefully contoured to improve definition and reduce dermatochalasis, maintaining symmetry. Eyelid tarsal exposure was avoided to prevent lagophthalmos. Following the upper eyelid procedure, a fully ablative and/or fractional Er:YAG laser, 2940 nm wavelength, (Sciton Inc., USA) was applied to the lower (and upper eyelid as necessary) eyelid skin for resurfacing. Patients received either a fully ablative laser or a fractional laser on the lower eyelids. The laser settings were adjusted based on skin thickness, patient skin type, downtime desired by the patient, and patient comfort. Fully ablative settings varied from 80 μm ablation with 50 μm coagulation for one or two passes to 40 μm ablation with 70 μm coagulation for one or two passes. Fractional erbium on the lids included 125–175 μm depth with Level 1–3 coagulation and 11%–22% density. Settings on the lower lid were customized for each patient within the above parameters. Patients with Fitzpatrick skin types 3–5 received fractional erbium on the lower lid. Patients with healthy and adequately thick lower eyelid skin, Fitzpatrick skin types 1–2, and downtime got fully ablative erbium on their lower eyelids. In general, patients who needed more tightening due to significant laxity got 40 μm ablation with 70 μm coagulation. On the other hand, if a patient needed more collagen due to crepey skin and sun damage, they got 80 μm ablation with 50 μm coagulation.

For postoperative care, patients were monitored postoperatively and petrolatum jelly was placed along the upper suture line as well as lower eyelid area. Instructions included avoiding sun exposure and applying petrolatum 3 times daily to protect the treated area. Follow‐up evaluations were scheduled at 1 day, 1 week for suture removal, 1 month, and 3 months post‐procedure to assess healing and results.

The artificial intelligence algorithm used in this study was developed by Kesty AI (Kesty AI, Florida, USA) to evaluate patient photographs and predict the Fitzpatrick Wrinkle Scale and Glogau Wrinkle Scale. The patient photographs were uploaded to the Kesty AI website (https://www.kesty.ai) and put into the proprietary algorithm. The machine learning model produced a rating for each photograph on both the Fitzpatrick Wrinkle Scale and the Glogau Wrinkle Scale. The machine learning algorithm used was developed based on thousands of patient photographs that were evaluated by a Dermatologist. These ratings were then “taught” to a machine learning model. This model was rigorously tested for validity to accurately predict patient characteristics based on a picture of the face. Characteristics that the artificial intelligence model can predict include the Fitzpatrick Wrinkle Scale and the Glogau Wrinkle Scale, which were used in this study. A machine learning algorithm is preferred over human evaluation in research because of the standardization of results. This can help reduce human error in evaluating before and after laser and other cosmetic enhancements.

## 3 Results

**FIGURE 1:**
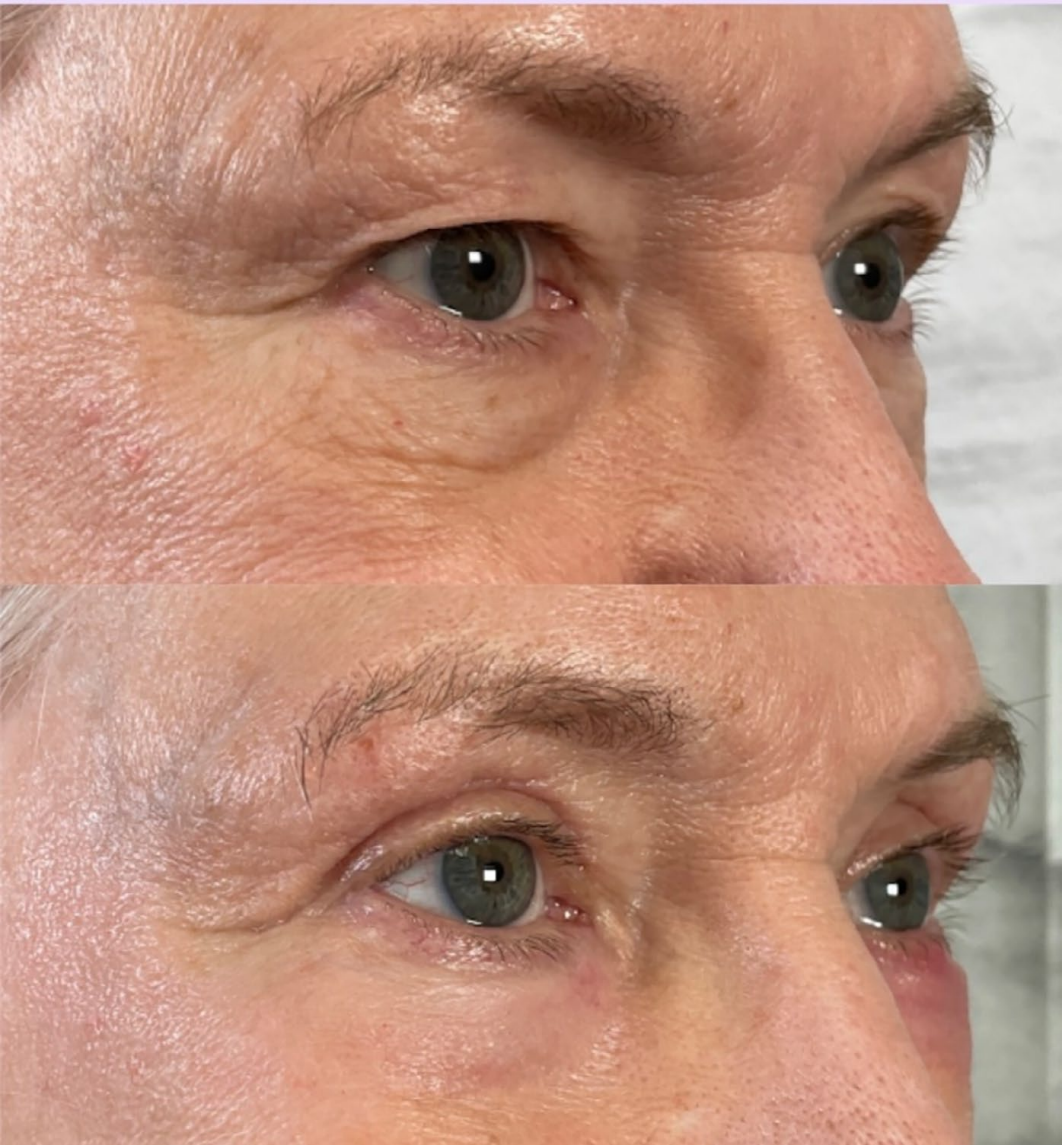
Patient before and 3 months after a combined upper CO_2_ blepharoplasty and Erbium laser resurfacing procedure.

**FIGURE 2:**
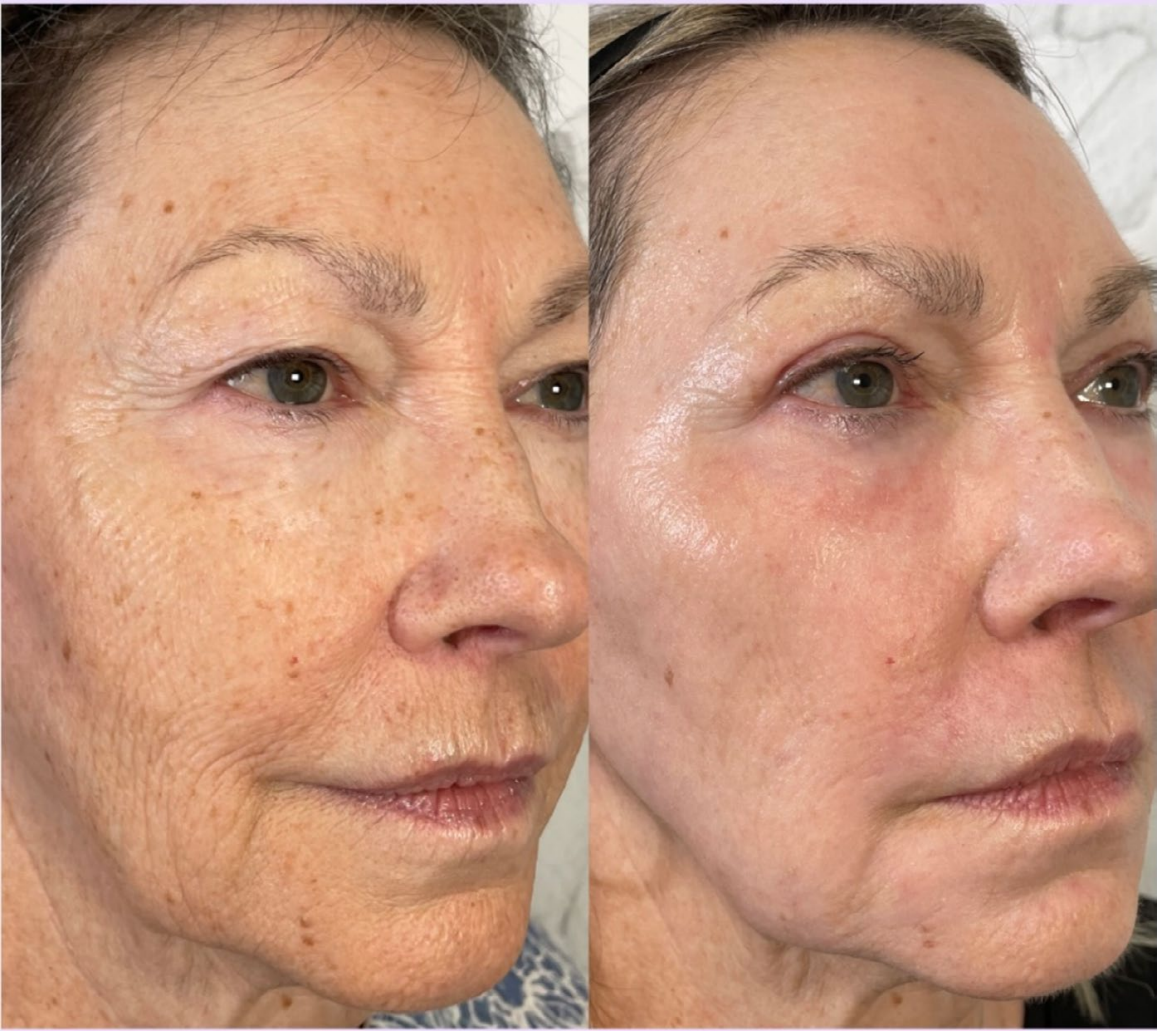
Patient before and 1 month after a combined upper CO_2_ blepharoplasty and Erbium laser resurfacing procedure.

**FIGURE 3:**
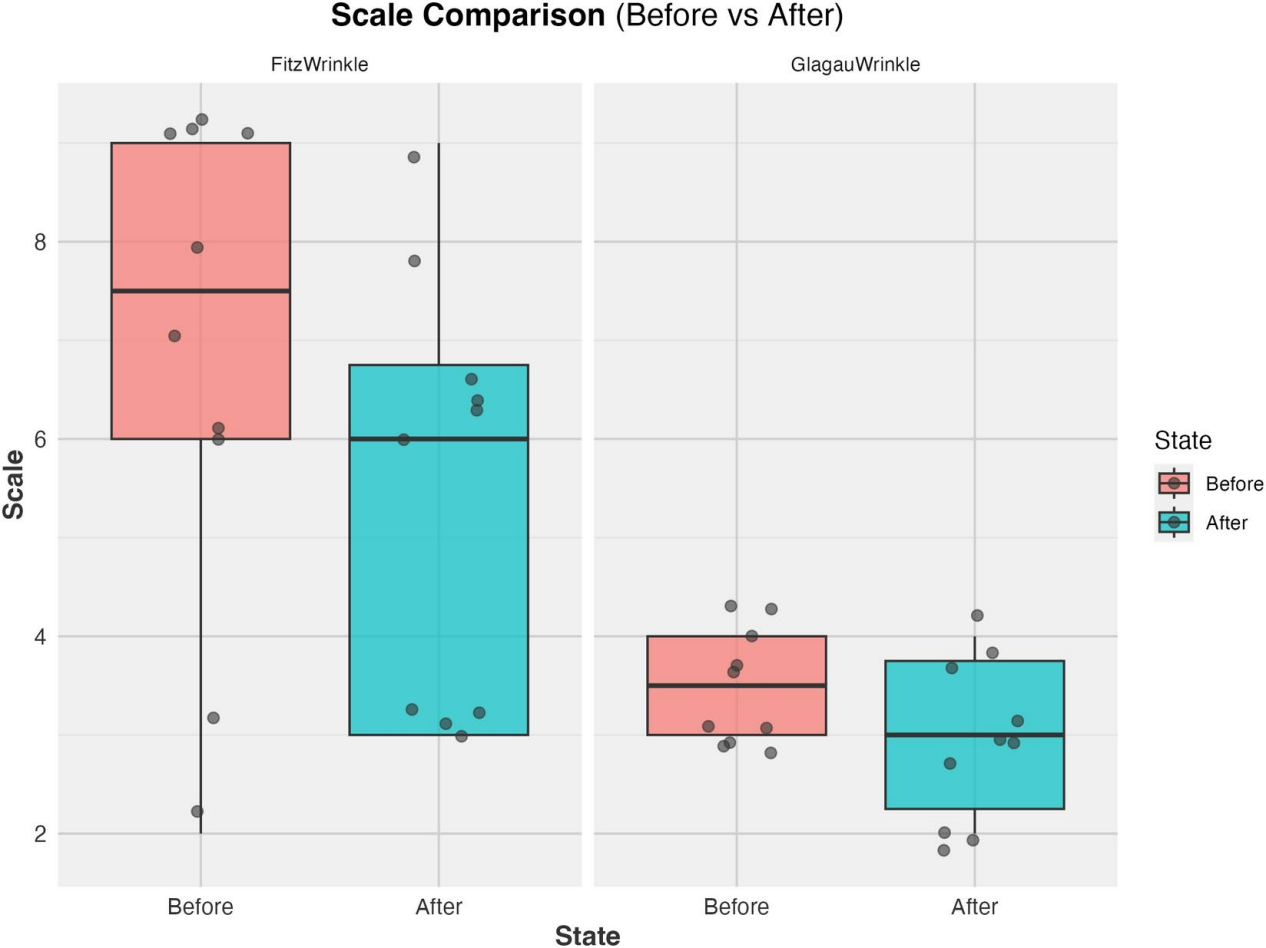
Artificial intelligence algorithm used to compare before and after results of the CO_2_ and Erbium laser blepharoplasty and eye rejuvenation, comparing Fitzpatrick Wrinkle Scale and Glogau Wrinkle Scale on 20 photographs.

**FIGURE 4:**
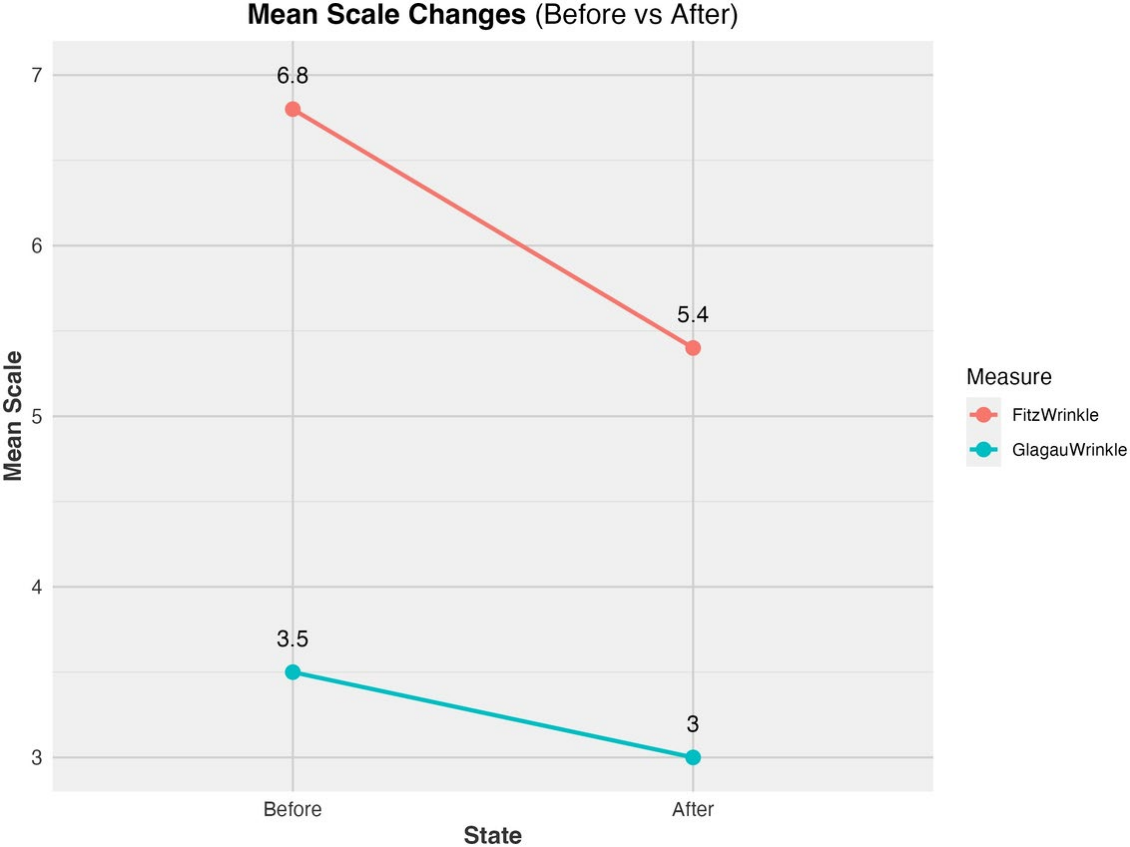
Mean scale changes using artificial intelligence large language models for the Fitzpatrick Wrinkle Scale and Glogau Wrinkle Scale to quantify the results of a laser procedure of CO_2_ blepharoplasty and erbium resurfacing for eye rejuvenation.

All patients tolerated the procedure well, with minimal discomfort reported and transient postoperative erythema lasting 4–10 days. No adverse events, such as ectropion, infection, hypopigmentation, or excessive scar tissue, were noted. By the 1‐month follow‐up, patients demonstrated significant improvements in upper eyelid contour, reduced skin laxity, and smoother lower eyelid texture. Patient satisfaction was high, with each patient reporting an overall rejuvenated appearance and a more “awake” and youthful look (Figures 1 and 2). Using the artificial intelligence algorithm, the average Fitzpatrick Wrinkle Scale of the 10 before photographs was a 6.8 (median 7.5). The average Fitzpatrick Wrinkle Scale of the 10 after photographs was a 5.4 (median 6.0) (Figure 3). The average Glogau Wrinkle Scale measurement using the artificial intelligence model was a 3.5 before the procedure (median 3.5) and decreased to 3.0 (median 3.0) after the laser (Figure 4).

## 4 Discussion

The combination of CO_2_ laser blepharoplasty and Er:YAG laser resurfacing addresses both upper eyelid dermatochalasis and lower eyelid wrinkles effectively while minimizing recovery time and the potential for complications.

The combined approach of CO_2_ laser blepharoplasty for the upper eyelids and Er:YAG laser resurfacing for the lower eyelids leverages the strengths of both modalities. Using the CO_2_ laser for the upper eyelid allows for precision in skin removal and hemostasis, while Er:YAG in the lower eyelid minimizes thermal injury and post‐inflammatory hyperpigmentation risks.

Post‐treatment recovery is typically shorter than traditional blepharoplasty. Patients in this series experienced only mild erythema and swelling, resolving within days to weeks. No long‐term complications were reported, suggesting that CO_2_ and Er:YAG lasers, when applied appropriately, can achieve appropriate eyelid rejuvenation.

Lasers have been studied extensively for facial skin rejuvenation, and the use of carbon dioxide laser for achieving a cosmetic blepharoplasty has been reported [3, 4, 5, 15, 16]. Furthermore, previous studies corroborate that the combined use of CO_2_ and Er:YAG lasers provides satisfactory outcomes with reduced recovery times and low complication rates [17]. Existing literature highlights the use of both carbon dioxide and erbium laser modalities separately for eye rejuvenation [14]. To our knowledge, this is the first report to present a combination of carbon dioxide upper blepharoplasty and erbium eyelid resurfacing on the same day for patients.

The results of the artificial intelligence model to quantify the positive results in this study showing a decrease in both the Fitzpatrick Wrinkle Scale and the Glogau Wrinkle Scale are in line with the significantly positive clinical results. The use of artificial intelligence algorithms and models to quantify results of laser procedures is a novel addition to the literature. Further studies could add this component to their results to eliminate the human error involved in evaluating results by a study participant.

## 5 Conclusion

This case series demonstrates that combining CO_2_ laser blepharoplasty for the upper eyelids with Er:YAG laser resurfacing for the lower eyelids is a viable option for achieving periorbital rejuvenation. This approach offers an alternative to traditional surgical blepharoplasty, with high patient satisfaction, minimal downtime, and a favorable safety profile.

## Author Contributions

K.R.K. and C.E.K. conceived the study, developed the laser protocols and settings, wrote and revised the manuscript, and funded the study. All authors have reviewed and approved the article for submission.

## Conflicts of Interest

K.R.K. is the Founder of Kesty AI, which was used in this paper.

## Data Availability

The data that support the findings of this study are available on request from the corresponding author. The data are not publicly available due to privacy or ethical restrictions.
